# Detection and Characterization of Viral Pathogens Associated with Reproductive Failure in Wild Boars in Central Italy

**DOI:** 10.3390/ani11020304

**Published:** 2021-01-25

**Authors:** Maria Irene Pacini, Mario Forzan, Giovanni Cilia, Fabrizio Bertelloni, Filippo Fratini, Maurizio Mazzei

**Affiliations:** Department of Veterinary Sciences, University of Pisa, Viale delle Piagge 2, 56124 Pisa, Italy; mariairene.pacini@phd.unipi.it (M.I.P.); mario.forzan@unipi.it (M.F.); giovanni.cilia@vet.unipi.it (G.C.); fabrizio.bertelloni@unipi.it (F.B.); filippo.fratini@unipi.it (F.F.)

**Keywords:** suid alphaherpesvirus 1, porcine circovirus 2, porcine parvovirus 1, wild boar foetus, pregnant sow

## Abstract

**Simple Summary:**

Suid herpesvirus 1, porcine circovirus 2 and porcine parvovirus are causative agents of reproductive failures in swine and are widely diffused in the wild boar population. No data describing the impact of those viruses on the reproductive performance of wild boar are so far available. We aimed to investigate the ability of the above viruses to infect foetuses of free-ranging pregnant wild boar sows living in a highly-populated area. Molecular investigation revealed that although all investigated viruses were detected in pregnant sows, only herpesvirus and circovirus were detected in the foetuses. Phylogenetic analysis revealed a close relationship between the strains circulating in wild boar and those already described in domestic swine. This study highlights the importance of monitoring the circulation of pathogens that are shared between domestic and wild pigs. This information is essential for the pig industry to avoid possible economic losses.

**Abstract:**

Wild boar and domestic swine share several pathogens, including viruses responsible for reproductive failures, representing an important sanitary and economic risk for the swine industry. Among them, suid herpesvirus 1 (SuHV-1), porcine circovirus 2 (PCV2) and porcine parvovirus 1 (PPV1) are widely diffused in the wild boar population. Unfortunately, little is known about their pathogenetic mechanisms and impact on the reproductive parameters of wild animals. This study aims to investigate the presence of viruses responsible for reproductive failure in pregnant wild boar sows and their foetuses. The investigation was conducted on 46 pregnant wild boar and their foetuses by molecular analysis; a phylogenetic study was performed on the positive samples. All of the investigated pathogens were identified in sows, while only herpesvirus and circovirus were detected in the tissues of their foetuses. Phylogenetic analysis revealed that the viral sequences obtained from the positive wild boars were closely related to those previously identified in domestic swine belonging to the same study areas. The results suggest that SuHV-1 and PCV2 can infect wild boar foetuses, with a possible impact on wild boar reproductive performance. Moreover, our data highlight the importance of continuous monitoring of swine pathogens circulating in wild environments, so as to carry out adequate sanitary actions.

## 1. Introduction

Wild boar (*Sus scrofa*) is one of the most widely distributed ungulates, characterized by a highly adaptable capability, a high reproductive rate and the ability to assume an opportunistic feeding behaviour [1]. For these peculiarities, its number and distribution are constantly increasing [2]. In Europe, the consistency of the wild boar populations is generally high, including in many Italian regions, and often including suburban areas [2].

Wild boar and domestic swine belong to the same species and microorganism transmission between them often occurs, especially in pigs bred in extensive or semi-extensive farms [3]. In an ecosystem, the presence of numerous available hosts and a high contact rate are factors that could lead to increased spread of infectious diseases, either among the wild boars themselves or between them and domestic animals. Furthermore, the role of wild boar as a possible reservoir for several pathogens could represent a severe risk for the wild and domestic animals’ health [4,5].

Considering that among the Suidae pathogens an important portion is represented by viral agents responsible for reproductive diseases, such as suid herpesvirus 1 (SuHV-1), porcine parvovirus 1 (PPV1) and circovirus 2 (PCV2), an investigation about the presence of those infectious agents in the wild boar population is rather important.

Swine herpesvirus 1 (SuHV-1) is a member of the family *Herpesviridae*, subfamily Alphaherpesvirinae and genus Varicellovirus, responsible for Pseudorabies (PR) or Aujeszky’s disease, a globally distributed infection of domestic and feral swine [6]. Suidae are the natural hosts of SuHV-1, in which the virus establishes a lifelong latent infection, which can be fatal in other host species [7,8].

In a high animal density environment, SuHV-1 is mainly transmitted by oro-nasal secretions, while venereal transmission has been identified as an alternative transmission route in feral swine [6,9,10].

In pregnant sows, the infection, as well as the reactivation of the virus, leads to Stillbirth, Mummification, Embryo Death and Infertility (SMEDI), depending on the month in which the virus reaches the placenta [11,12,13,14]. Therefore, SuHV-1 is of great impact on the swine industry, forcing the implementation of coordinated eradication programs [15], mainly based on large-scale vaccination of farmed pigs by a gE-deleted vaccine [6]. Despite the important goal reached, the circulation of SuHV-1 in wild swine is still present in several European countries, including Italy, where serological prevalence ranges from 4% to 66% [6,10,15,16,17,18,19,20,21,22,23]. No data are available on the reproductive effect of the wild boar population.

Porcine circoviruses are a group of small viruses belonging to the *Circoviridae* family. Four species have been described: the non-pathogenic PCV1; the most diffused porcine circovirus 2 (PCV2); porcine circovirus type 3 (PCV3), recently identified in domestic swine and wild boar; and porcine circovirus type 4 (PCV4) [24,25,26,27,28].

PCV2 is an important and ubiquitous pathogen of domestic swine with seroprevalence reaching almost 100%. It is responsible for ‘‘porcine circovirus diseases’’ (PCVD) [29].

Several field studies have confirmed the vertical transmission of PCV2 to the foetus and its association with reproductive disorders, abortions, mummification and stillbirths, due to foetal viral replication during all pregnancy stages [30]. The foetal myocardium appears to be the preferred site of viral replication, resulting in severe myocarditis [31,32,33,34,35]. On the other hand, often the PCV2 foetal infection does not lead to PCV2-associated reproductive disease and the intrauterine-infected piglets can be clinically normal [36,37,38]. This clinical difference is likely related to the timing of foetal PCV2 infection (late gestation) and the degree of PCV2 replication [37]. Wild boar can also be infected by PCV2 and can suffer from PMWS [39,40,41,42,43,44,45,46]. In Europe, the seroprevalence in wild boar is high, ranging from 23% to 58% [39,40,43,46,47,48,49], reaching a value of 39.8% in Italy [50]. Unfortunately, no available data are present about the reproductive PCV2 impact on the wild boar population.

Porcine parvovirus (PPV) belongs to the genus Parvovirus, the family *Parvoviridae*. Among domestic pigs, the virus has a worldwide distribution and it is endemic in most herds [51]. Eight different phylogenetic groups of parvoviruses have been identified from pigs, including PPV1, PPV2, PPV3, PPV4, PPV5, porcine bocaviruses (PBoV) and, recently, PPV6 and PPV7 [52,53].

In swine, PPV1 infection of susceptible pregnant sows can result in embryonic and foetal death, mummification and stillbirth, resulting in severe losses for the pig industry [51,54,55,56,57].

The clinical outcome of PPV1 in the foetus is strictly dependent on the time of gestation in which the virus infects the sow. An infection by PPV1 occurring during the first half of pregnancy can lead to reproductive failure, while foetuses infected after Day 70 of gestation can develop an antibody response and often survive the infection [51,58,59].

The severity of reproductive failure depends on the virulence of the PPV1 strains. Indeed, highly pathogenic strains (e.g., Kresse and 27a) cross the placental barrier more efficiently than low pathogenic and vaccine strains (e.g., NADL-2 and MSV) [60,61,62].

PPV1 is widely distributed in the wild boar population of Europe, with high seroprevalence values ranging from 30 to 78% [48,63,64,65,66,67,68].

Studies conducted on the wild boar populations have shown that PPV1 is also present in Italy, with a prevalence ranging from 8% to 99% according to the study areas [69,70,71].

However, despite the strong evidence of PPV1 circulating in wild boar, there is little information on the effects of the virus on wild boar health and reproductive performance; although, according to a study by Ruiz-Fons and colleagues, it seems to be associated with a decrease in the ovulation rate in female wild boar [64].

In domestic swine, SuHV-1, PCV2 and PPV1can be transmitted from pregnant sows to foetuses with several consequences on pregnancy or the piglets’ health. Due to the wildness of wild boar and the difficulty to monitor their reproductive performance and parturitions, limited information is available about the pathogenesis of SuHV-1, PCV2 and PPV1 in pregnant wild boar sows and about their ability to infect foetuses, with effects on the course of pregnancy.

This study aims to investigate the ability of the main causative viral agents of reproductive failure in swine to infect foetuses in free-ranging pregnant wild boar sows living in a highly-populated area.

## 2. Materials and Methods

### 2.1. Sample Collection

During the 2018–2019 and 2019–2020 hunting seasons, from 1 November to 31 January, tissue samples were collected from pregnant wild boar hunted in Tuscany (Italy) in a specific area that constitutes contiguous municipalities (Pisa, Siena, Grosseto and Livorno province), known for the copious presence of wild animals. The animals were hunted following the Regional Hunting Law (Regolamento di attuazione della legge regionale 12 gennaio 1994 no. 3 DPGR 48/R/2017). Lymph nodes and foetal specimens were sampled from 26 animals during the slaughtering procedures. The lymph nodes were sampled directly from carcasses of the animals while the pregnant uteruses were conveyed to the Department of Veterinary Science (University of Pisa) for foetus sampling. Foetuses were weighed and measured to retrieve information about their development stage, then tissue samples of the heart, lung, liver, kidney and spleen, belonging to all foetuses, were collected from a single sow and pooled for molecular analysis.

### 2.2. Molecular Analysis

Each lymph node and the foetus samples were subjected to tissue disruption (Tissue Lyser Qiagen, Hilden, Germany) before the DNA extraction was performed, using the DNeasy Blood and Tissue kit (Qiagen, Hilden, Germany).

Molecular assays were performed individually for all DNAs using PCR protocols designed to identify the SuHV-1, PPV1 and PCV2 genomes [72,73,74].

A first set of highly sensitive PCRs was applied for diagnostic purposes to identify the positive sample; further sets of PCRs were performed to obtain the phylogenetic information from all the positive samples.

Samples that were positive after the molecular analysis were submitted to sequence analysis (BMR genomics, Padova, Italy).

In Table 1 the primer sets used for the molecular analysis are presented.

### 2.3. Phylogenetic Analysis

Nucleotide sequence analysis was applied to confirm the specificity of the PCR assays and to obtain phylogenetic information on the viral strains circulating in the studied areas. For each viral target investigated, a set of the most representative GenBank available sequences were identified and used to construct phylogenetic trees by maximum-likelihood methods, as available in the MEGA6 software package [76]. Phylogenetic analysis for SuHV-1 was conducted on 404 positions of the gE gene in the final dataset, for PCV2 on 431 positions of Open Reading Frame 2 (ORF2) and for PPV1 on 776 positions of Viral Protein 2 (VP2). The evolutionary history was inferred by using the Maximum Likelihood method based on the Tamura–Nei model.

The bootstrap test was applied to calculate the percentage of replicate trees in which the associated taxa clustered together (100 replicates).

## 3. Results

Concerning foetus sampling, they resulted in a weight median value of 169 (±71.1) grams with a median length of 138.6 (±19.3). Due to the collection sample period, the common seasonal wild boar mating season and the dimension of the collected foetuses, we can assume that the age of foetuses sampled was in the range of 50 to 70 days of gestation [77].

All the viral agents studied were found in at least one sow. The results of this investigation indicated that 1 out of 26 pregnant wild boars was positive for PCV2, 2 out of 26 were positive for parvovirus and 1 out of 26 was double-positive for SuHV-1 and PPV1.

Foetal samples collected from the pregnant wild boar positive for SuHV-1 and PPV1 were positive for SuHV-1, but not for PPV1. In addition, the foetal samples from the PPV1-positive sow were negative for the same virus. Furthermore, the pooled foetuses sampled from the PCV2-positive sow were also positive (Table 2). The foetuses belonging to negative wild boar were negative for all studied pathogens.

All positive results were confirmed by sequence analysis. Moreover, a 100% nucleotide sequence identity was detected, comparing sows and their foetuses positive for SuHV-1 and PCV2.

**Figure 1:**
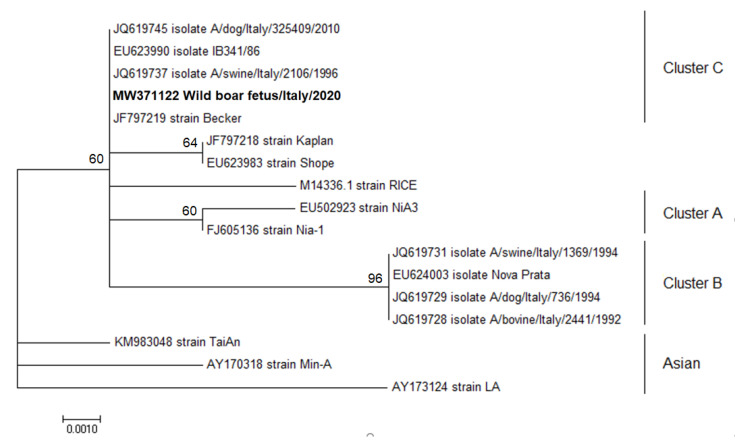
Molecular phylogenetic analysis by Maximum Likelihood method for gE of SuHV-1. The evolutionary history was inferred using the Maximum Likelihood method based on the Tamura–Nei model. The percentage of replicate trees in which the associated taxa clustered together in the bootstrap test (100 replicates) is shown next to the branches. The tree with the highest log likelihood (−627.94) is shown. The analysis involved 17 nucleotide SuHV-1 sequences with a total of 404 positions for gE gene in the final dataset. Evolutionary analyses were conducted in MEGA6 [76]. GenBank accession numbers are shown when the available host, state and year of GenBank sequences are presented. The sequence identified in the present work is represented in bold characters.

Results obtained from the phylogenetic analyses performed on the single viral pathogen investigated demonstrated that the SuHV-1 strain detected in pregnant wild boar and associated foetuses (based on gE gene) was identical to a herpesvirus strain previously detected in 1996 in Italy from swine, and a strain isolated from a dog in 2010 belonging to Cluster C (Figure 1).

**Figure 2:**
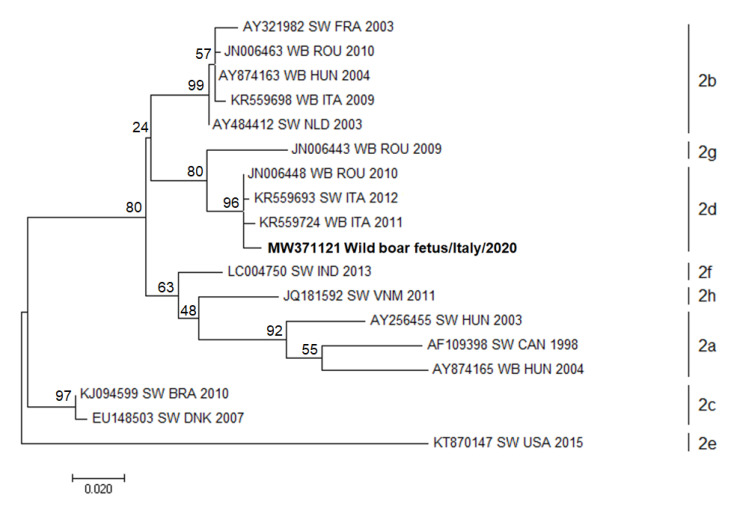
Molecular phylogenetic analysis by Maximum Likelihood method for PCV2. The evolutionary history was inferred by using the Maximum Likelihood method based on the Tamura–Nei model. The percentage of replicate trees in which the associated taxa clustered together in the bootstrap test (100 replicates) is shown next to the branches. The tree with the highest log likelihood (−1839.33) is shown. The analysis involved 18 nucleotide PCV2 sequences with a total of 431 positions for PCV2 in the final dataset. Evolutionary analyses were conducted in MEGA6 [76]. GenBank accession numbers are shown when the available host, state and year of GenBank sequences are presented, and classified in genotypes (2a-2h). The sequence identified in the present work is represented in bold characters. SW: swine; FRA: France; WB: wild boar; ROU: Romania; HUN: Hungary; ITA: Italy; NDL: Netherlands; IND: India; VNM: Vietnam; CAN: Canada; BRA: Brazil; DNK: Denmark; USA: Unite States of America.

A similar result, showing a correlation to previous sequences identified in Italy, was observed for swine circovirus. In this case, the Italian wild boar sequence identified in the present study was closely related to sequences derived from wild boar and domestic swine collected in Italy in 2011 and 2012, respectively. The phylogenetic analysis classifies the PCV2 sequences as belonging to PCV2d (Figure 2).

**Figure 3:**
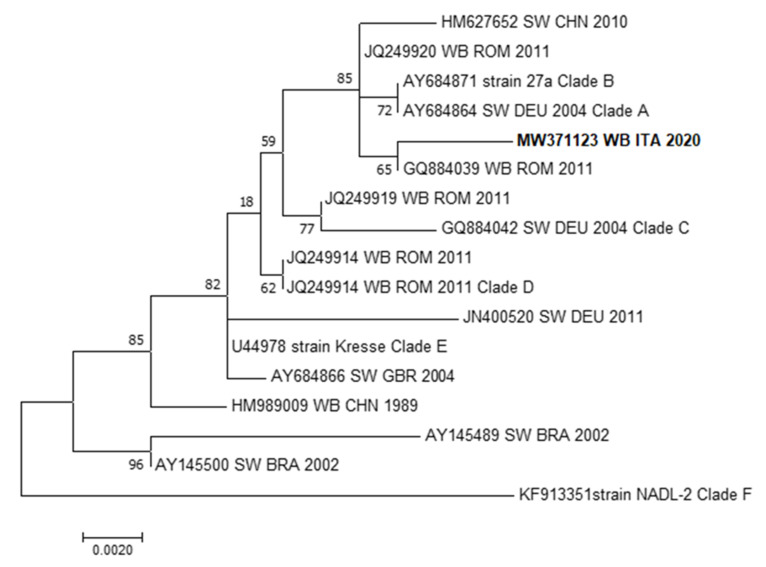
Molecular phylogenetic analysis by Maximum Likelihood method for PPV1. The evolutionary history was inferred by using the Maximum Likelihood method based on the Tamura–Nei model. The percentage of replicate trees in which the associated taxa clustered together in the bootstrap test (100 replicates) is shown next to the branches. The tree with the highest log likelihood (−1384.63) is shown. The analysis involved 17 nucleotide PPV1 sequences with a total of 776 positions for the PPV1-VP2 gene in the final dataset. Evolutionary analyses were conducted in MEGA6 [76]. GenBank accession numbers are shown when the available host, state and year of GenBank sequences are presented. The sequence identified in the present work is represented in bold characters. WB: wild boar; ROU: Romania; SW: swine; CHN: China; ITA: Italy; DEU: Germany; GBR: Great Britain; BRA: Brazil.

Finally, the parvovirus sequence identified from two pregnant wild boars showed a complete homology with each other and correlates with a PPV1 wild boar sequence from Romania collected in 2011, as indicated by the VP2 sequence analysis (Figure 3).

## 4. Discussion

Numerous studies previously conducted on the Italian wild boar population have revealed the presence of several pathogens, and among them, the viral agents responsible for reproductive disorders [10,15,46,70,78,79,80]. The present research has highlighted the circulation of SuHV-1, PCV2, and PPV1 in wild boar circulating in a research area located in central Italy. The area is characterized by a high wild boar population density in which hunting activity is widely diffused. By molecular assays, in this research, we identified the presence of all the pathogens studied, confirming their long-time persistence in the Italian wild boar population. Moreover, the identification of the genome of those viral agents in the tissue samples indicates their active circulation and has allowed us to conduct phylogenetical analysis.

The results described in the present work highlight the importance of wild boar testing for monitoring the presence of infectious diseases in a certain ecosystem. Unfortunately, due to the limited number of samples, no epidemiological information about prevalence could be inferred.

All the pathogens investigated were detected in at least one pregnant sow and SuHV-1 and PCV2 were also detected in the foetus tissues, confirming their ability to infect foetuses during the first stage of gestation. In particular, the SuHV-1 positivity has confirmed the results obtained in a previous research study conducted in the same study area [79]. It is noteworthy that, in the present study, the SuHV-1-positive pregnant wild boar was positive also for PPV1. Moreover, a second PPV1-positive pregnant wild boar was detected in the same municipality (Grosseto province). Although the presumed gestation time of 50–70 days could be considered as a susceptible period for PPV1 infection, foetal samples collected from both the PPV1-positive wild boar scored negative in the molecular assay [51,59]. Probably, the negative results in the foetuses could be justified considering that the virus in domestic swine needs 12–18 days to reach the foetus after the mother becomes infected. Moreover, the mother’s immunity could be capable of protecting the foetuses from infection [51,58,59].

Finally, circovirus infection was identified in a sow and the associated foetuses, confirming the ability of the virus to cross the placenta.

For all positive cases, no macroscopic clinical evidence was recorded, neither for the adults during the standard slaughter procedures nor for foetuses during sample preparation.

The phylogenetic analysis performed on the sequences obtained for the positive samples belonging to each viral pathogen investigated revealed a close relationship to the previously detected Italian strains, confirming the continuous circulation of such viral types among the Italian wild boar population, and often with high homology to domestic animals.

In detail, the SuHV-1 gE sequences analysed cluster with suid herpesvirus Cluster C, confirming previous phylogenetic studies that identified Clusters B and C as the most diffused among domestic swine in Italy. This evidence highlights that the SuHV-1 viral type circulating in domestic swine are currently circulating also in feral animals [80].

Concerning PCV2, studies using phylogenetic analysis defined eight different genotypes of PCV2 (PCV2a to PCV2h), of which PCV2a, b and d are the most common around the world. In Italy, PCV2b is prevalent at the moment, but the PCV2d frequency is progressively rising following a stronger genotype shift from PCV2b to PCV2d, which started in 2010 and now reported on a worldwide scale [81,82,83]. The results are perfectly in line with the phenomenon described in the literature since the phylogenetic analysis of the obtained sequences identifies the detected PCV2 as belonging to Genotype D.

Concerning PPV1, the phylogenetic analysis can provide little information since the parvoviral genes are highly conserved and no PPV1 wild boar sequences are available from Italy. More recent studies revealed that the virus could be divided into distinct clusters based on some amino acid substitutions on the VP1/VP2 genes. Moreover, few residue substitutions in the VP1/VP2 proteins can lead different virus strains to different tissue tropism, virulence and pathogenetic patterns. Consequently, there are some low-virulence strains of PPV1 (i.e., NADL-2), some moderately virulent strains and some highly virulent strains (e.g., Kresse, 27a strains) [74]. The results of the phylogenetic analysis indicate an association of our sequence with the highly virulent strains. This finding should raise questions about the impact on the swine industry related to pathogen transmission from wild to domestic swine.

## 5. Conclusions

In conclusion, this work evidenced the presence and circulation of three of the most important viral agents responsible for reproductive failures in swine in wild boar in Italy. The information about the reproductive impact of such viral agents on the wild boar population is still scarce due to the difficulties in identifying the negative impact on reproduction in a wild species with a high reproductivity rate. This finding suggests that SuHV-1 and PCV2, responsible for reproductive failures in domestic swine, can maintain the same tropism for foetal tissues in wild boar. This evidence could be useful to get additional knowledge about the reproductive performance in feral swine. Noteworthy, in the studied area the presence of wild boar is abundant, but few swine industries are present. However, swine breeding is mainly based on an extensive rearing system, and animals living outdoors have the possibility to frequently encounter wild animals.

Therefore, continuous monitoring of the health status of the wild boar population is important to monitor the presence of circulating pathogens, to provide sanitary indications.

## Figures and Tables

**Table 1:** The primers used in the PCRs, type of virus, type of PCR assay, target gene, primer sequence, expected product and references.

Virus	PCR Assay	Target	Primer Sequence (5′–3′)	Expected Product (bp)	References
PCV2	Diagnostic and Phylogenetic	ORF2	Fw: CGGATATTGTAGTCCTGGTCG Rw: ACTGTCAAGGCTACCACAGTC	481	Giammarioli et al., 2008 [73]
PPV1	Diagnostic	VP2	Fw: GCAGTACCAATTCATCTTCT Rw: TGGTCTCCTTCTGTGGTAGG	158	Giammarioli et al., 2008 [73]
	Phylogenetic	VP1	Fw: ACCAACCTGCACTTAACTCC Rw: GTGTGTGTGCATCGTCTTGT	970	Cadar et al., 2012 [74]
	Phylogenetic	VP1/VP2	Fw: GAGGTAAGAAGATCG CCGAG Rw: TCCTACCTGAGCTGGCCTAA	1136	
	Phylogenetic	VP2	Fw: CT ACCACAGAAGGAGACCAA Rw: ATTGAAGTATACAATGATAGTAGT	928	
SuHV-1	Diagnostic Nested	gB	Fw1: ATGGCCATCTCGCGGTGC Rw1: ACTCGCGGTCCTCCAGCA	334	Yoon et al., 2005 [72]
			Fw2: ACGGCACGGGCGTGATC Rw2: GGTTCAGGGTACCCCGC	195	
	Phylogenetic	gE	Fw: CCGCGGGCCGTGTTCTTTGT Rw: CGTGGCCGTTGTGGGTCAT	500	Huang et al., 2004 [75]

**Table 2:** Comparison of the PCR results among the pregnant sows and pooled foetuses (number of pooled foetuses), year and municipality of the positive samples.

Sample Year Municipality	Sample Type	PCV2	PPV1	SuHV-1
WB.1091 2019 Grosseto	Pregnant sow	−	+	+
	Pooled foetuses (3)	−	−	+
WB.111 2019 Grosseto	Pregnant sow	−	+	−
	Pooled foetuses (4)	−	−	−
WB.211 2019 Lucca	Pregnant sow	+	−	−
	Pooled foetuses (1)	+	−	−

## References

[1] Gethöffer F., Sodeikat G., Pohlmeyer K. (2007). Reproductive Parameters of Wild Boar (*Sus scrofa*) in Three Different Parts of Germany. Eur. J. Wildl. Res..

[2] Pittiglio C., Khomenko S., Beltran-Alcrudo D. (2018). Wild Boar Mapping Using Population-Density Statistics: From Polygons to High Resolution Raster Maps. PLoS ONE.

[3] Miller R., Sweeney S., Slootmaker C., Reports D.G.-S. (2017). Cross-Species Transmission Potential between Wild Pigs, Livestock, Poultry, Wildlife, and Humans: Implications for Disease Risk Management in North America. Sci. Rep..

[4] Ruiz-Fons F., Segalés J., Gortázar C. (2008). A Review of Viral Diseases of the European Wild Boar: Effects of Population Dynamics and Reservoir Rôle. Vet. J..

[5] Meng X.J., Lindsay D.S. (2009). Wild Boars as Sources for Infectious Diseases in Livestock and Humans. Philos. Trans. R. Soc. B: Biol. Sci..

[6] Müller T., Hahn E.C., Tottewitz F., Kramer M., Klupp B.G., Mettenleiter T.C., Freuling C. (2011). Pseudorabies Virus in Wild Swine: A Global Perspective. Arch.Virol..

[7] Enquist L.W. (1994). Infection of the Mammalian Nervous System by Pseudorabies Virus (PRV). Semin. Virol..

[8] Ruiz-Fons F., Vidal D., Höfle U., Vicente J., Gortazar C. (2007). Aujeszky’s Disease Virus Infection Patterns in European Wild Boar. Vet. Microbiol..

[9] Romero C.H., Meade P.N., Shultz J.E., Chung H.Y., Gibbs E.P., Hahn E.C., Lollis G. (2001). Venereal Transmission of Pseudorabies Viruses Indigenous to Feral Swine. J. Wildl. Dis..

[10] Verin R., Varuzza P., Mazzei M., Poli A. (2014). Serologic, Molecular, and Pathologic Survey of Pseudorabies Virus Infection in Hunted Wild Boars (*Sus scrofa*) in Italy. J. Wildl. Dis..

[11] Wittmann G., Rziha H.-J., Knipe D.M., Howley P.M. (1989). Aujeszky’s Disease (Pseudorabies) in Pigs. Herpesvirus Diseases of Cattle, Horses and Pigs.

[12] Papageorgiou K.V., Burriel A.R., Filioussis G., Psychas V., Nauwynck H.J., Kritas S.K. (2011). Aujeszky’s Disease (Pseudorabies). An Old Threat in Current Pig Industry? Part I. Pathogenetic Information and Implications. J. Hell. Vet. Med. Soc..

[13] Yu X., Zhou Z., Hu D., Zhang Q., Han T., Li X., Gu X., Yuan L., Zhang S., Wang B. (2014). Pathogenic Pseudorabies Virus, China, 2012. Emerg. Infect. Dis..

[14] Dors E.C., Mól M.P. (2017). Aujeszky’s disease. Emerging and Re-Emerging Infectious Diseases of Livestock.

[15] Moreno A., Sozzi E., Grilli G., Gibelli L., Lavazza A., Cordioli P. (2015). Detection and Molecular Analysis of Pseudorabies Virus Strains Isolated from Dogs and a Wild Boar in Italy. Vet. Microbiol..

[16] Caruso C., Vitale N., Prato R., Radaelli M.C., Zoppi S., Possidente R., Dondo A., Chiavacci L., Maria A., Martin M. (2018). Pseudorabies Virus in North-West Italian Wild Boar (*Sus scrofa*) Populations: Prevalence and Risk Factors to Support a Territorial Risk-Based Surveillance. Vet. Ital..

[17] José M. (2002). Antibodies to Selected Viral and Bacterial Pathogens in European Wild Boars from Southcentral Spain. Wildl. Dis. Assoc..

[18] Vicente J., Ruiz-Fons F., Vidal D., Hofle U., Acevedo P., Villanua D., Fernandez-De-Mera I.G., Martin M.P., Gortazar C. (2005). Serosurvey of Aujeszky’s Disease Virus Infection in European Wild Boar in Spain. Vet. Rec..

[19] Vengust G., Valencak Z., Bidovec A. (2005). Presence of Antibodies Against Aujeszky’s Disease Virus in Wild Boar (*Sus scrofa*) in Slovenia. Wildl. Dis. Assoc..

[20] Denzin N., Conraths F.J., Mettenleiter T.C., Freuling C., Müller T. (2020). Monitoring of Pseudorabies in Wild Boar of Germany—A Spatiotemporal Analysis. Pathogens.

[21] Pannwitz G., Freuling C., Denzin N., Schaarschmidt U., Nieper H., Hlinak A., Burkhardt S., Klopries M., Dedek J., Hoffmann L. (2012). A Long-Term Serological Survey on Aujeszky’s Disease Virus Infections in Wild Boar in East Germany. Epidemiol. Infect..

[22] Cano-Manuel F., López-Olvera J., Granados J. (2014). Long-Term Monitoring of 10 Selected Pathogens in Wild Boar (*Sus scrofa*) in Sierra Nevada National Park, Southern Spain. Vet. Microbiol..

[23] Lari A., Lorenzi D., Faccini S. (2006). Pseudorabies virus in european wild boar from central Italy. Source J. Wildl. Dis..

[24] Collins P.J., McKillen J., Allan G. (2017). Porcine Circovirus Type 3 in the UK. Vet. Rec..

[25] Klaumann F., Correa-Fiz F., Franzo G., Sibila M., Núñez J.I., Segalés J. (2018). Current Knowledge on Porcine Circovirus 3 (PCV-3): A Novel Virus with a yet Unknown Impact on the Swine Industry. Front. Vet. Sci..

[26] Klaumann F., Dias-Alves A., Cabezón O., Mentaberre G., Castillo-Contreras R., López-Béjar M., Casas-Díaz E., Sibila M., Correa-Fiz F., Segalés J. (2019). Porcine Circovirus 3 Is Highly Prevalent in Serum and Tissues and May Persistently Infect Wild Boar (*Sus scrofa scrofa*). Transbound. Emerg. Dis..

[27] Franzo G., Ruiz A., Grassi L., Sibila M., Drigo M., Segalés J. (2020). Lack of Porcine Circovirus 4 Genome Detection in Pig Samples from Italy and Spain. Pathogens.

[28] Zhang H., Hu W., Li J., Liu T., Zhou J., Opriessnig T., Xiao C. (2020). Novel Circovirus Species Identified in Farmed Pigs Designated as Porcine Circovirus 4, Hunan Province, China. Transbound. Emerg. Dis..

[29] Allan G.M., Ellis J.A. (2000). Porcine Circoviruses: A Review. J. Vet. Diagn. Invest..

[30] Harding J.C.S. (2004). The Clinical Expression and Emergence of Porcine Circovirus 2. Proc. Vet. Microbiol..

[31] West K.H., Bystrom J.M., Wojnarowicz C., Shantz N., Jacobson M., Allan G.M., Haines D.M., Clark E.G., Krakowka S., McNeilly F. (1999). Myocarditis and Abortion Associated with Intrauterine Infection of Sows with Porcine Circovirus. J. Vet. Diagn. Investig..

[32] Kim J., Jung K., Chae C. (2004). Prevalence of Porcine Circovirus Type 2 in Aborted Fetuses and Stillborn Piglets. Vet. Rec..

[33] Brunborg I.M., Jonassen C.M., Moldal T., Bratberg B., Lium B., Koenen F., Schönheit J. (2007). Association of Myocarditis with High Viral Load of Porcine Circovirus Type 2 in Several Tissues in Cases of Fetal Death and High Mortality in Piglets. A Case Study. J. Vet. Diagn. Investig..

[34] Madson D.M., Patterson A.R., Ramamoorthy S., Pal N., Meng X.J., Opriessnig T. (2009). Reproductive Failure Experimentally Induced in Sows via Artificial Insemination with Semen Spiked with Porcine Circovirus Type 2. Vet. Pathol..

[35] Rose N., Opriessnig T., Grasland B., Jestin A. (2012). Epidemiology and Transmission of Porcine Circovirus Type 2 (PCV2). Virus Res..

[36] Pensaert M., Sanchez R.E., Ladekjær-Mikkelsen A.S., Allan G.M., Nauwynck H.J. (2004). Viremia and Effect of Fetal Infection with Porcine Viruses with Special Reference to Porcine Circovirus 2 Infection. Vet. Microbiol..

[37] Madson D.M., Opriessnig T. (2011). Effect of Porcine Circovirus Type 2 (PCV2) Infection on Reproduction: Disease, Vertical Transmission, Diagnostics and Vaccination. Anim. Health Res. Rev. Conf. Res. Work. Anim. Dis..

[38] Segalés J. (2012). Porcine Circovirus Type 2 (PCV2) Infections: Clinical Signs, Pathology and Laboratory Diagnosis. Virus Res..

[39] Toplak I., Grom J., Hostnik P., Barlič-Maganja D. (2004). Phylogenetic Analysis of Type 2 Porcine Circoviruses Indentified in Wild Boar in Slovenia. Vet. Rec..

[40] Vicente J., Segalés J., Höfle U., Balasch M., Plana-Durán J., Domingo M., Gortázar C. (2004). Epidemiological Study on Porcine Circovirus Type 2 (PCV2) Infection in the European Wild Boar (*Sus scrofa*). Vet. Res..

[41] Knell S., Willems H., Hertrampf B., Reiner G. (2004). Comparative Genetic Characterization of Porcine Circovirus Type 2 Samples from German Wild Boar Populations. Vet. Microbiol..

[42] Corrêa A.M.R., Zlotowski P., Rozza D.B., Borba M.R., Leal J.D.S., da Cruz C.E.F., Driemeier D. (2006). Postweaning Multisystemic Wasting Syndrome in Farmed Wild Boars (*Sus scrofa*) in Rio Grande Do Sul. Pesqui. Vet. Bras..

[43] Cságola A., Kecskeméti S., Kardos G., Kiss I., Tuboly T. (2006). Genetic Characterization of Type 2 Porcine Circoviruses Detected in Hungarian Wild Boars. Arch. Virol..

[44] Lipej Z., Segalés J., Jemeršić L., Olvera A., Roić B., Novosel D., Mihaljević Ž., Manojlović L. (2007). First Description of Postweaning Multisystemic Wasting Syndrome (PMWS) in Wild Boar (*Sus scrofa*) in Croatia and Phylogenetic Analysis of Partial PCV2 Sequences. Acta Vet. Hung..

[45] Sofia M., Billinis C., Psychas V., Birtsas P., Sofianidis G., Leontides L., Knowles N., Spyrou V. (2008). Detection and genetic characterization of porcine circovirus 2 isolates from the first cases of postweaning multisystemic and wasting syndrome in wild boars in greece. Source J. Wildl. Dis..

[46] Morandi F., Verin R., Sarli G., Canetti N., Scacco M., Panarese S., Poli A. (2010). Porcine Circovirus Type 2 (PCV2) Antigen Localisation and Post-Weaning Multisystemic Wasting Syndrome (PMWS) in Free-Ranging Wild Boar (*Sus scrofa* Ssp scrofa) in Italy. Eur. J. Wildl. Res..

[47] Sedlak K., Bartova E., Machova J. (2008). Antibodies to Selected Viral Disease Agents in Wild Boars from the Czech Republic. Wildl. Dis. Assoc..

[48] Closa-Sebastià F., Casas-Díaz E., Cuenca R., Lavín S., Mentaberre G., Marco I. (2011). Antibodies to Selected Pathogens in Wild Boar (*Sus scrofa*) from Catalonia (NE Spain). Eur. J. Wildl. Res..

[49] Reiner G., Bronnert B., Hohloch C., Fresen C., Haack I., Willems H., Reinacher M. (2010). Qualitative and Quantitative Distribution of PCV2 in Wild Boars and Domestic Pigs in Germany. Vet. Microbiol..

[50] Delogu M., Ostanello F., Martin A.M., Lelli D., Frasnelli M., Marzadori F., Raffini E., De Marco M.A. Infezione da PCV2 nel cinghiale: Dinamica anticorpale in una popolazione monitorata in un’area protetta (2002–2006). Proceedings of the IV Workshop Nazionale di Epidemiologia Veterinaria.

[51] Mengeling W.L., Lager K.M., Vorwald A.C. (2000). The Effect of Porcine Parvovirus and Porcine Reproductive and Respiratory Syndrome Virus on Porcine Reproductive Performance. Anim. Reprod. Sci..

[52] Miłek D., Woźniak A., Stadejek T. (2018). The Detection and Genetic Diversity of Novel Porcine Parvovirus 7 (PPV7) on Polish Pig Farms. Res. Vet. Sci..

[53] Miłek D., Woźniak A., Guzowska M., Stadejek T. (2019). Detection Patterns of Porcine Parvovirus (PPV) and Novel Porcine Parvoviruses 2 through 6 (PPV2–PPV6) in Polish Swine Farms. Viruses.

[54] Cutlip R.C., Mengeling W.L. (1975). Pathogenesis of in Utero Infection: Experimental Infection of Eight- and Ten-Week-Old Porcine Fetuses with Porcine Parvovirus. Am. J. Vet. Res..

[55] Mengeling W.L., Lager K.M., Zimmerman J.K., Samarikermani N., Beran G.W. (1991). A Current Assessment of the Role of Porcine Parvovirus as a Cause of Fetal Porcine Death. J. Vet. Diagn. Investig..

[56] Soares R.M., Durigon E.L., Bersano J.G., Richtzenhain L.J. (1999). Detection of Porcine Parvovirus DNA by the Polymerase Chain Reaction Assay Using Primers to the Highly Conserved Nonstructural Protein Gene, NS-1. J. Virol. Methods.

[57] Pérez L.J., Díaz de Arce H., Percedo M.I., Domínguez P., Frías M.T. (2010). First report of porcine circovirus type 2 infections in Cuba. Res. Vet. Sci..

[58] Joo H., Donaldson-Wood C.R., Johnson R. (1976). Observations on the Pathogenesis of Porcine Parvovirus Infection. Arch. Virol..

[59] Mengeling W.L., Paul P.S., Brow T.T. (1980). Transplacental Infection and Embryonic Death Followinq Maternal Exposure to Porcine Parvovirus Near the Time of Conception. Arch. Virol..

[60] Kresse J.I., Taylor W.D., Stewart W.W., Eernisse K.E.-V. (1985). Parvovirus Infection in Pigs with Necrotic and Vesicle-like Lesions. Vet. Microbiol..

[61] Zeeuw E.J.L., Leinecker N., Herwig V., Selbitz H.J., Truyen U. (2007). Study of the Virulence and Cross-Neutralization Capability of Recent Porcine Parvovirus Field Isolates and Vaccine Viruses in Experimentally Infected Pregnant Gilts. J. Gen. Virol..

[62] Mészáros I., Olasz F., Cságola A., Tijssen P., Zádori Z. (2017). Biology of Porcine Parvovirus (Ungulate Parvovirus 1). Viruses.

[63] Roic B., Jemersic L., Terzic S., Keros T., Balatinec J., Florijancic T. (2012). Prevalence of Antibodies to Selected Viral Pathogens in Wild Boars (*Sus scrofa*) in Croatia in 2005–06 and 2009–10. J. Wildl. Dis..

[64] Ruiz-Fons F., Vicente J., Vidal D., Höfle U., Villanúa D., Gauss C., Segalés J., Almería S., Montoro V., Gortázar C. (2006). Seroprevalence of Six Reproductive Pathogens in European Wild Boar (*Sus scrofa*) from Spain: The Effect on Wild Boar Female Reproductive Performance. Theriogenology.

[65] Vengust G., Valencak Z., Bidovec A. (2006). A Serological Survey of Selected Pathogens in Wild Boar in Slovenia. J. Vet. Med. Ser. B: Infect. Dis. Vet. Public Health.

[66] Lelešius R., Sereika V., Zienius D., Michalskienė I. (2006). Serosurvey of wild boar population for porcine parvovirus and other selected infectious diseases in Lithuania. Bull. Vet. Inst. Pulawy..

[67] Kaden V., Lange E., Hänel A., Hlinak A., Mewes L., Hergarten G., Irsch B., Dedek J., Bruer W. (2009). Retrospective Serological Survey on Selected Viral Pathogens in Wild Boar Populations in Germany. Eur. J. Wildl. Res..

[68] Malmsten A., Magnusson U., Ruiz-Fons F., González-Barrio D., Dalin A.M. (2018). A Serologic Survey of Pathogens in Wild Boar (*Sus scrofa*) in Sweden. J. Wildl. Dis..

[69] Cordioli P., Callegari S., Berlinzani A., Foni E., Candotti P., Barigazzi G. (1993). Indagine sierologica su cinghiali selvatici dell’Appennino parmense (Serological survey in wild boars from “Appennino parmense” area). Atti. Soc. Ital. Sci. Vet..

[70] Ercolini C., Ferrari A., Fisichella S., Guerci L.P., Mandola M.L., Masoero L., Mignone W., Perruchon M., Poggi M. (2014). Serological Survey of Wild Boar (*Sus scrofa*) in Liguria, Italy. J. Mt. Ecol..

[71] Fenati M., Armaroli E., Corrain R., Guberti V. (2009). Indirect Estimation of Porcine Parvovirus Maternal Immunity Decay in Free-Living Wild Boar (*Sus scrofa*) Piglets by Capture-Recapture Data. Vet. J..

[72] Yoon H.A., Eo S.K., Aleyas A.G., Park S.O., Lee J.H., Chae J.S., Cho J.G., Song H.J. (2005). Molecular Survey of Latent Pseudorabies Virus Infection in Nervous Tissues of Slaughtered Pigs by Nested and Real-Time PCR. Artic. J. Microbiol..

[73] Giammarioli M., Pellegrini C., Casciari C., de Mia G.M. (2008). Development of a Novel Hot-Start Multiplex PCR for Simultaneous Detection of Classical Swine Fever Virus, African Swine Fever Virus, Porcine Circovirus Type 2, Porcine Reproductive and Respiratory Syndrome Virus and Porcine Parvovirus. Vet. Res. Commun..

[74] Cadar D., Dán Á., Tombácz K., Lorincz M., Kiss T., Becskei Z., Spînu M., Tuboly T., Cságola A. (2012). Phylogeny and evolutionary genetics of porcine parvovirus in wild boars. Infect. Genet. Evol..

[75] Huang C., Hung J.J., Wu C.Y., Chien M.S. (2004). Multiplex PCR for rapid detection of pseudorabies virus, porcine parvovirus and porcine circoviruses. Vet. Microbiol..

[76] Tamura K., Stecher G., Peterson D., Filipski A., Kumar S. (2013). MEGA6: Molecular Evolutionary Genetics Analysis Version 6.0. Mol. Biol. Evol..

[77] Henry V.G. (1968). Fetal Development in European Wild Hogs. J. Wildl. Manag..

[78] Dei Giudici S., lo Presti A., Bonelli P., Angioi P.P., Sanna G., Zinellu S., Balzano F., Salis F., Ciccozzi M., Oggiano A. (2019). Phylogenetic Analysis of Porcine Circovirus Type 2 in Sardinia, Italy, Shows Genotype 2d Circulation among Domestic Pigs and Wild Boars. Infect. Genet. Evol..

[79] Pacini M.I., Forzan M., Cilia G., Bernardini L., Marzoli F., Pedonese F., Bandecchi P., Fratini F., Mazzei M. (2020). Detection of Pseudorabies Virus in Wild Boar Foetus. Animals.

[80] Sozzi E., Moreno A., Lelli D., Cinotti S., Alborali G.L., Nigrelli A., Luppi A., Bresaola M., Catella A., Cordioli P. (2014). Genomic Characterization of Pseudorabies Virus Strains Isolated in Italy. Transbound. Emerg. Dis..

[81] Franzo G., Segalés J. (2018). Porcine Circovirus 2 (PCV-2) Genotype Update and Proposal of a New Genotyping Methodology. PLoS ONE.

[82] Franzo G., Tinello S., Grassi L., Tucciarone C.M., Legnardi M., Cecchinato M., Dotto G., Mondin A., Martini M., Pasotto D. (2020). Free to Circulate: An Update on the Epidemiological Dynamics of Porcine Circovirus 2 (PCV-2) in Italy Reveals the Role of Local Spreading, Wild Populations, and Foreign Countries. Pathogens.

[83] Franzo G., Cortey M., Segalés J., Hughes J., Drigo M. (2016). Phylodynamic analysis of porcine circovirus type 2 reveals global waves of emerging genotypes and the circulation of recombinant forms. Mol. Phylogenetics Evol..

